# Narrowly distributed taxa are disproportionately informative for conservation planning

**DOI:** 10.1038/s41598-021-03119-9

**Published:** 2022-02-09

**Authors:** Munemitsu Akasaka, Taku Kadoya, Taku Fujita, Richard A. Fuller

**Affiliations:** 1 Faculty of Agriculture, Tokyo University of Agriculture and Technology, Fuchu, Tokyo 183-8509 Japan; 2 School of Biological Sciences, The University of Queensland, St. Lucia, QLD 4072 Australia; 3 Institute of Global Innovation Research, Tokyo University of Agriculture and Technology, Fuchu, Tokyo 183-8509 Japan; 4 Center for Environmental Biology and Ecosystem Studies, National Institute for Environmental Studies, Tsukuba, Ibaraki 305-8506 Japan; 5 Graduate School of Comprehensive Human Sciences, University of Tsukuba, Tsukuba, Ibaraki 305-8571 Japan; 6 The Nature Conservation Society of Japan, 1-16-10, Shinkawa, Chuo-ku, Tokyo 104-0033 Japan

**Keywords:** Conservation biology, Conservation biology

## Abstract

Biological atlas data can be used as inputs into conservation decision-making, yet atlases are sometimes infrequently updated, which can be problematic when the distribution of species is changing rapidly. Despite this, we have a poor understanding of strategies for efficiently updating biological atlas data. Using atlases of the distributions of 1630 threatened plant taxa, we quantitatively compared the informativeness of narrowly distributed and widespread taxa in identifying areas that meet taxon-specific conservation targets, and also measured the cost-efficiency of meeting those targets. We also explored the underlying mechanisms of the informativeness of narrowly distributed taxa. Overall, narrowly distributed taxa are far more informative than widespread taxa for identifying areas that efficiently meet conservation targets, while their informativeness for identifying cost-efficient areas varied depending on the type of conservation target. Narrowly distributed taxa are informative mainly because their distributions disproportionately capture areas that are either relatively taxon rich or taxon poor, and because of larger number of taxa captured with given number of records. Where resources for updating biological data are limited, a focus on areas supporting many narrowly distributed taxa could benefit conservation planning.

## Introduction

Data on the distribution of biodiversity ultimately underpin many conservation decisions, and the process of collecting such data, particularly occurrence records, itself consumes substantial conservation resources^1^. Major atlas projects can serve as vehicles for spatial data collection, but such atlases are often only rarely updated^2^, regardless of their spatial extent (i.e., national or regional). For example, major editions of the British flora were published in 1962 and 2002^3^, of Australian birds in 1984 and 2002^4^, of Japanese birds in the 1970s and 1990s^5^, and of breeding birds of New York state in 1980s and 2000s^6^.

While such major atlas efforts periodically yield comprehensive data, surveys during the intervening period are often (although not always) less structured, less well resourced and/or have limited spatial coverage, e.g.^7,8^. Careful targeting of those surveys, such as biasing them toward the geographic ranges of narrowly distributed taxa, could yield rapid improvements in the utility of the data for conservation planning. Indeed, it is generally well known that data on narrowly distributed taxa are more informative, or in other words provide more information to identify priority areas, than widely distributed taxa for efficient conservation planning^9–11^, but whether programs acquiring biodiversity data should explicitly target narrowly distributed taxa with this in mind depends on the magnitude of the difference in their informativeness^12^. When the difference is large, targeting survey effort toward regions known to contain narrowly distributed taxa could usefully inform conservation planning even when available resources are limited. Kujala et al.^13^ showed that narrowly distributed taxa strongly shape the identification of priority conservation areas, but it remains unclear whether only including narrowly distributed taxa in a prioritisation will also result in efficient area selection for more widespread taxa that are not considered during the prioritization.

Here we quantitatively compare the relative importance of distribution data on narrowly and widely distributed taxa for identifying efficient conservation networks, and explore the underlying mechanisms that lead to the informativeness of data on narrowly distributed taxa. That concept of informativeness of taxa shares an underlying conceptual similarity with irreplaceability^14^, although the former is a measure of taxa while the latter is a measure of a location. We use comprehensive atlas data on the spatial distribution of 1630 threatened vascular plant taxa in Japan, and measure the relative informativeness of data on narrowly distributed taxa in constructing conservation plans compatible with Target 7 of the Global Strategy for Plant Conservation (GSPC https://www.cdb.int/gspc/), which states that “At least 75 per cent of known threatened plant taxa [are] conserved in situ by 2020”. Our aim was to provide a theoretical basis for prioritizing species for surveying when updating atlas data aimed at identifying conservation priority areas.

## Results

**Figure 1:**
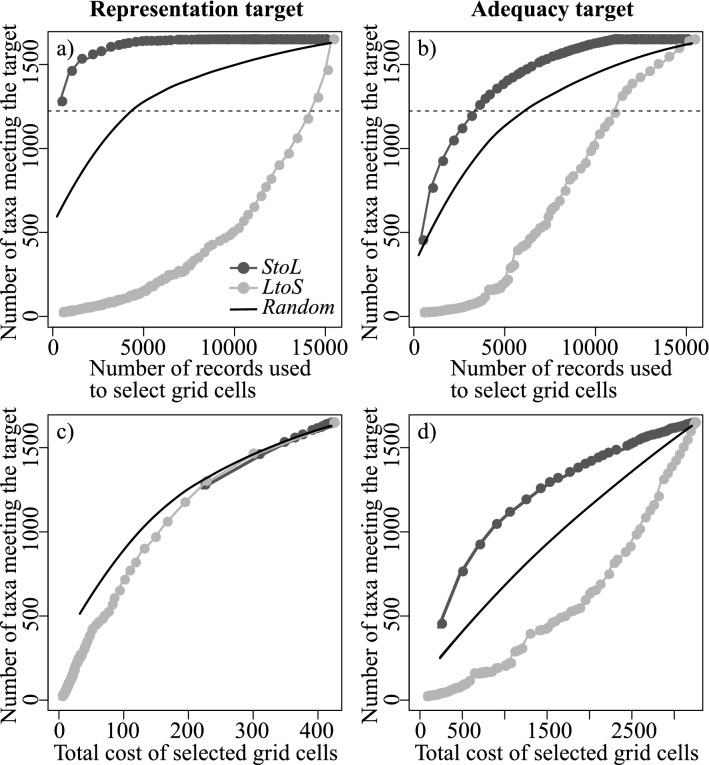
(**a**, **b**) Relationship between the number of records used to select grid cells (*x*-axis) and the number of taxa meeting conservation targets (*y*-axis) using taxa distribution data sequentially from small to large (*StoL*: dark grey), large to small (*LtoS*; light grey) or a random order (black). For the random order, the relationship was displayed by drawing a lowess smoothing line of the 50 iterations together with 2SE error bounds (although error bounds are too narrow to visualize). (**c**, **d**) Relationship between total cost of selected grid cells (*x*-axis) and the number of taxa meeting the target (*y*-axis) for *StoL* (dark grey), *LtoS* (light grey) and random (black). Results for representation targets (i.e., representing one grid for each taxa) (**a**, **c**) and adequacy targets (i.e., representing 100% of the distribution for taxa with AOO ≤ 50 grid cells, and representing 50 grid cells for taxa with the larger AOO) (**b**, **d**). Horizontal dashed lines indicate 75% of the overall taxa used (1223 taxa).

Under the *StoL* strategy, only 2.2% (338 records) of the 15,304 distribution records, constituting 338 taxa, were necessary to identify conservation networks that achieved the representation target (representing one grid for each taxa) of 75% (i.e. target 7 of GSPC) for all threatened taxa in the dataset (Fig. 1a). In stark contrast, 94.3% (14,432 records, 1025 taxa) and 28.2% of records (4323 records, 450 taxa) were required to achieve the same target under the *LtoS* or *random* strategy, respectively. The superiority of the *StoL* strategy in identifying areas meeting the GSPC target also remained when adequacy targets (representing 100% of the distribution for taxa with AOO ≤ 50 grid cells, and representing 50 grid cells for taxa with larger AOO) were used instead of a proportion-based representation target [23.2% (3457 records, 1183 taxa), 73.8% (11,299 records, 382 taxa), 39.7% (6081 records, 648 taxa), respectively for *StoL*, *LtoS*, and *random*; Fig. 1b).

Overall, *StoL* outperformed *LtoS* by 144.0% and 210.6% in increasing the number of taxa meeting the target per number of records used to select conservation grid cells, respectively for adequacy targets and the representation target (Fig. 1a,b, see AUC of each strategy for Supplementary Information 3). Notably, *random* generally outperformed *LtoS*.

The number of taxa meeting the adequacy target with *StoL* increased more quickly with increasing cost than it did with *LtoS*. (RABC = 1.440, Fig. 1d). In contrast, change in the number of taxa meeting the representation target as the total cost of selected grid cells increased was similar among the three orderings (RABC = 0.972, Fig. 1c). This suggests that narrowly distributed taxa are far more informative for identifying areas that efficiently improve the number of taxa meeting conservation targets for a given number of records used regardless of the type of target set, while their informativeness in identifying cost-efficient areas does depend on the type of target.

**Figure 2:**
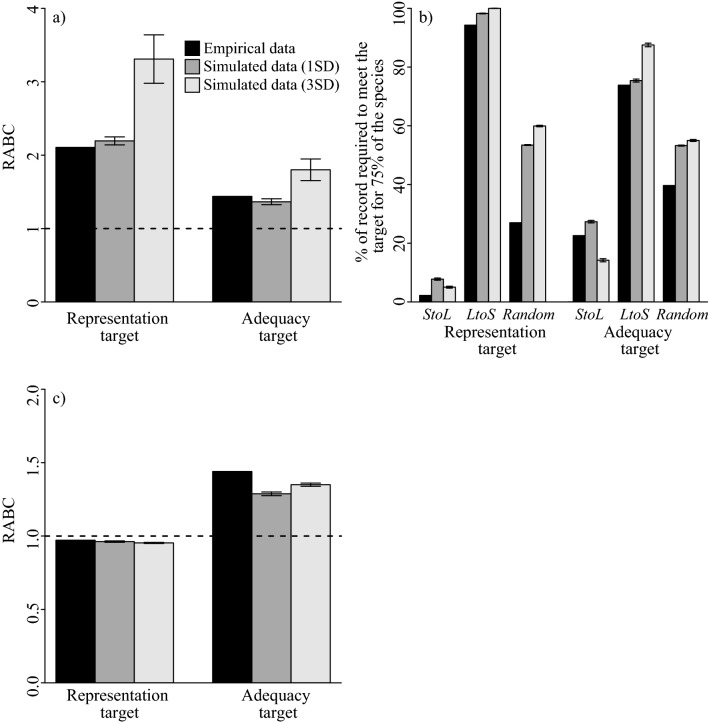
The performance of *StoL* over *LtoS* on randomly generated taxon distributions in a 68 × 68 square space. Performance was evaluated by (**a**) the number of taxa meeting the target, (**b**) the percentage of records required to meet the conservation target of 75% of the taxa being protected, and (**c**) the total cost of selected grid cells.

The degree of informativeness of *StoL* over *LtoS* (i.e. RABCs and percentage of number of records required to meet the conservation target of 75% of the taxa) for the virtual taxa generated based on the empirical standard deviation (simulated data 1SD) was similar to that of the empirical data regardless of the target and metric used to evaluate the informativeness (Fig. 2), suggesting that the observed pattern in the empirical data is independent of the pattern of overlap among taxa and the shape of the study region. In the similar vein, the degree of informativeness of *StoL* for virtual taxa generated based on wider standard deviation (simulated data 3SD) was similar to that for empirical data, except higher performance on the number of taxa meeting the target for representation target (Fig. 2), suggesting that the superior informativeness of narrowly distributed taxa is maintained even if a species pool contains some very widespread species.

**Figure 3:**
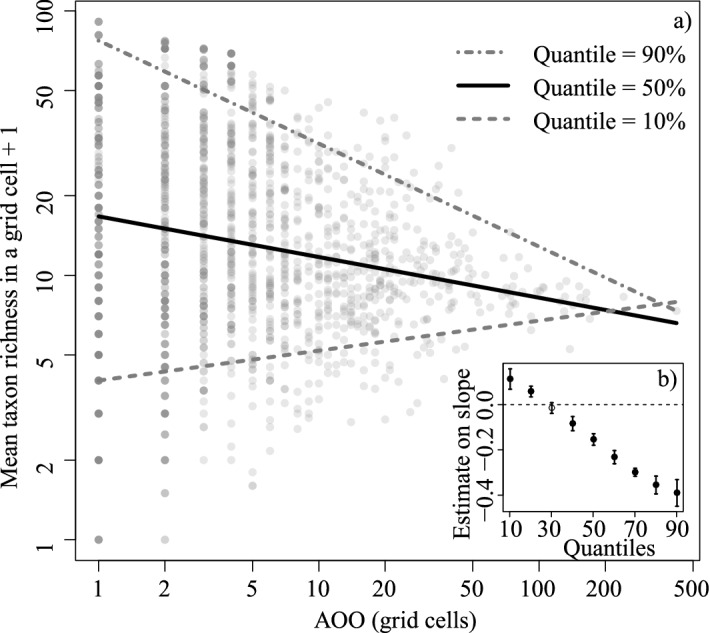
(**a**) Relationship between mean taxon richness in a grid cell and taxon AOO on Japanese threatened plants (empirical data). The black line denotes the predicted relationship based on 50th percentile regression, and grey dash, and dotted dash line denotes 10th and 90th percentile regression lines, respectively. (**b**) Change in the slope of the relationship along percentiles. Points and error bars indicate the estimate and 1.96 × Standard error. See also Supplementary Information 6 for estimates of the regressions.

Mean richness across a taxon’s range decreased as the taxon’s AOO increased, when the relationship was assessed at ≥ 40th percentile of the taxa richness variable (Fig. 3). Contrastingly, the relationship was significantly positive at ≤ 20th percentile (Fig. 3, Supplementary Information 6).

## Discussion

Our results show that a distribution record of a narrowly distributed taxon is markedly more informative than a record of a widely distributed taxon for identifying reserve networks that efficiently meet conservation targets, regardless of the type of conservation target set (Figs. 1, 2). These results agree with studies testing informativeness at a species level^9–11,15,16^, and those showing that a limited portion of distribution data is sufficient to identify efficient sets of areas for biodiversity conservation^13,17^. Further, we found that the efficient sets of areas for biodiversity conservation identified based only on distribution data of narrowly distributed taxa also sufficiently represent more widely distributed taxa that were not considered during the area selection. The difference in performance among alternative methods of ordering taxa was particularly evident at the initial stage of adding new distribution records. This suggests that the choice of data updating strategy, e.g. preferential updating of records of narrowly distributed taxa (*StoL* strategy), widespread taxa (*LtoS* strategy), or disregarding AOO (random/no strategy), will be particularly important when only a limited number of distribution records can be updated. This will be especially true in biological surveys for updating atlas data, in cases where data updating is extremely expensive or time consuming. In the case of Japanese threatened plants, only 2.2% of the overall distribution records (338 records) were sufficient to identify conservation networks that achieve the GSPC target 7 with at least one representation for each taxon when the *StoL* strategy was employed, while 43 fold (94.3%, 14,432 records) and 13 fold (28.2%, 4313 records) records were necessary when *LtoS* strategy or *random* strategy were adopted, respectively (Fig. 1). Moreover, the informativeness of the *StoL* strategy was maintained, and was nearly sevenfold more informative, even in the simulation based on randomly generated distribution records (Fig. 2). Our empirical analysis employed only threatened plants, however, we suggest the findings are applicable to wider taxonomic groups. This is because our empirical findings were consistent with results on the simulations with no assumptions specific to plants. Furthermore, the results of simulations in which virtual taxa had wider variation in AOO than the empirical data (i.e. virtual taxa generated based on a threefold standard deviation of the empirical approximation), suggests that the informativeness of narrowly distributed taxa could be applicable to species pools containing some very widespread species. Overall, where data updating resources are limited relative to the size of the task, our results suggest that updating of existing data could focus on those places where narrowly distributed taxa are known to occur. It would be fruitful to explore building predictive models that generate estimates of where narrowly distributed taxa might occur.

The *StoL* strategy outperformed *LtoS* by about 1.4-fold in identifying cost-effective areas to meet adequacy targets while controlling for the performance of *random* strategies, although not a representation target (Fig. 1). This suggests that narrowly distributed taxa are also informative for reducing the total cost of proposed conservation networks, and together these effects lead to enhanced overall informativeness to identify areas that meet taxon-specific conservation targets cost-efficiently, at least when using an adequacy target. Further, the informativeness of narrowly distributed taxa would become more evident over time through successive atlas updates, because conservation networks heavily influenced by uninformative taxa would likely require continued extension of conservation networks—as new taxa are recorded and need to be represented^18,19^. The performance of the *StoL* strategy under an adequacy target could also result from proportional targets requiring more land for very widespread taxa than very narrowly-distributed ones, e.g. 10% of the distribution of a very widespread taxon is likely to be a much greater land area than 100% of a very narrowly-distributed taxon. However, we found that after accounting for the amount of area needing to be selected, the *StoL* strategy still outperformed *LtoS* by 127.7% (Supplemental information 4), suggesting this effect is not large in our case.

The positive and negative relationships identified between taxon AOO and mean richness across a taxon’s range (Fig. 3) provide insight into the underlying mechanism driving the informativeness of narrowly distributed taxa other than the intuitive mechanism that the *StoL* strategy can deal with more species with a given number of records at the initial stage. The negative relationship between AOO and the mean richness across a taxon’s range indicates that, on average, taxa with a narrower AOO co-occur with more other taxa. The positive relationships on the 10th and 20th percentiles (Fig. 3b) suggest that many narrowly distributed taxa have little or no overlap with other taxa. Hence, narrowly distributed taxa comprise forms that have contrasting character: the majority intersect all taxa that occur in relatively taxa rich areas, while some occur in areas with very few co-occurring taxa, which are not captured when focusing search effort only on taxon rich areas. The relationship between taxon AOO and mean taxon richness was likewise positive and negative on randomly generated distribution data for virtual taxa used in the simulation (Supplementary Information﻿ 5). The relationship was positive when regressing the median, and negative when higher quantiles (i.e. 80, 90% percentiles) were regressed. Therefore, it seems likely that the contrasting properties rather than the positive median trend is more responsible for the high informativeness of narrowly distributed taxa.

Comprehensive distribution data are fundamental for a variety of conservation applications and ecological studies^20–22^, and we are certainly not suggesting ceasing such general atlasing efforts. However, with ongoing rapid loss of biodiversity and constraints in resources and time for updating data to identify conservation networks^1,23,24^, our results have important conceptual implications for updating atlas data to be used in prioritization schemes, particularly in regions where sampling is expensive or logistically difficult. We highlight the importance of using up-to-date distribution data to identify efficient conservation networks. If a comprehensive update cannot be achieved, our results imply that a partial update could be worthwhile if planned carefully, although a full value of information analysis would shed light on when it is most efficient to confirm existing records of rare species or to search for new ones. Our results imply that biasing resources for data collection first toward areas harboring species with very narrow AOO, using the atlas data itself to determine areas to survey. This is because these areas harbor either taxa with very narrow AOO and limited co-occurring taxa or many co-occurring taxa. Islands, mountain ranges, areas that have unusual environmental conditions or act as climatic refugia often harbor unique taxa, e.g.,^25–27^ and such areas could be candidate targets for surveys to update distribution records to identify and establish conservation areas.

The superior informativeness of narrowly distributed taxa we quantified is encouraging for conservation prioritization projects that use or plan to use atlas data compiled some time ago. In such projects, literature searches and citizen scientist collected records could effectively complement the existing atlas data, because both are known to be biased toward the locations of rare species, see^21,28^ respectively. The informativeness of narrowly distributed taxa turns what is often seen as a negative property into an asset for conservation-relevant monitoring. Citizen scientist collected data are also spatially biased in general, and so effective incentivisation of citizen scientists to survey regions with narrowly distributed taxa would contribute to maximizing efficiency in data updating, and by extension, conservation planning when resources are limited. Further, the current work serves as a starting point for identification of efficient set of areas for biodiversity monitoring to detect biodiversity loss, although considerations of a number of other factors would be essential for creating an implementable plan.

The spatial distribution of the portfolio of conservation priority is often largely influenced by the spatial distribution of cost, especially when species occurrence is positively associated with conservation cost in the landscape, or conservation cost is highly variable across the landscape^29^. Thereby, the degree of cost-efficiency for surveying narrowly distributed taxa, in terms of total cost of selected grid cells we quantified might be reduced if a focal area embraces very large variation in cost to reconduct surveys, and narrowly distributed taxa are predominantly distributed in the very high-cost units whereas widely distributed species occur mostly in low-cost units. Further investigation of the spatial dynamics of survey cost would be useful to help shape plans for updating atlases. Additionally, although we did not quantify time/overall cost saved by prioritizing atlas data to update in a real-world situation, such quantification would provide a firmer basis for those responsible for updating atlases.

Our argument on the superiority of narrowly ranged taxa over widely distributed ones is simple and intuitive, but we have been able to carefully quantify it. Rarity is determined not only by geographic range size and habitat specificity, which determine AOO at a coarse grain, but also by local abundance, an attribute independent from the former two^30^. Hence, when reliable estimates of local abundance are available, further improvement in efficiency might be achieved by weighting taxa by estimated local abundance. In practice, such consideration of local abundance might not typically be necessary, particularly when the organization that manages a data updating project outsources field survey, because local abundance of respective taxa would probably not correlate with the cost of surveillance.

## Methods

### Data

We used the latest distribution records of 1630 threatened vascular plants in Japan, originally collected to produce the updated 4th edition of the plant Red List of Japan (census conducted in 2010–2011 and released in 2012)^31,32^. Plant distribution data were collected across Japan during extensive surveys by more than 500 botanists organized by the Japanese Society for Plant Systematics (JSPS^33^), and organized into grid cells of approx. 10 km × 10 km. We only considered taxa (species, subspecies, and distinct varieties) categorized as Critically Endangered, Endangered, or Vulnerable in the 4th edition of the Red List, and taxa whose distribution was confirmed in the database among all the vascular plants recorded. We did not include Data Deficient taxa because it is difficult to capture entire distribution range, and Least Concern taxa because available dataset only includes few taxa. The available Least Concern taxa all had a smaller area of occurrence (AOO; < 340 grid cells) than taxa with largest AOO in the threatened species used in the analysis. We treated presence of a taxon in a grid cell as one distribution record in this study, because distribution records of each taxon were mostly originally collected at the scale of, or subsequently aggregated into, the grid cells. The dataset comprised 15,304 records, spanning 3119 of the 4654 grid cells covering Japanese terrestrial areas (excluding the northern territories). The mean and standard deviation (SD) of the area of occupancy (AOO) of focal taxa was 9.39 ± 21.03 grid cells, with the large SD being caused by the highly right skewed frequency distribution of the AOO (minimum and maximum range size were 1 grid and 421 grid cells, respectively; Supplementary Information 1). The mean and SD of number of taxa per grid cell was 4.91 ± 6.39 for grid cells harboring at least one taxon (minimum and maximum were 1 and 91, respectively).

### Data analysis

To assess the magnitude of informativeness of distribution records of narrowly distributed taxa over that of widely distributed taxa for meeting a conservation target, we conducted a Marxan analysis to efficiently select portfolios of grid cells that meet pre-defined conservation targets by adding data for taxa in forward (small to large; *StoL*) and reverse (large to small; *LtoS*) order of their AOO (see Supplementary Information 2 for the details of Marxan settings and calibration). Additionally to the sequential orderings of taxa, we randomly ordered the taxa and sequentially added groups of 50 taxa until all 1630 taxa were added, as a null model (*random*). Note that taxa captured by the selected portfolios include targeted taxa and non-targeted taxa. We assessed the relative performance of the former two sequences (i.e. *StoL* and *LtoS*) via three metrics, (1) the percentage of records required to meet a defined conservation target for 75% (i.e. 1223 taxa) of all the taxa used, (2) the number of taxa meeting one of the two considered conservation targets (i.e., representation target and adequacy target, see below) per record used, and (3) the number of taxa meeting each conservation target per the total cost of selected grid cells. We used two kinds of conservation targets, reflecting two common objectives of conservation planning. First, a representation target was considered achieved if any portion of the taxon’ distribution was included in a conservation network. Second, an adequacy target was considered achieved for a taxon where 100% of its occupied grid cells were included in a conservation network where the taxon’ AOO is ≤ 50 grid cells, and if 50 grid cells were included in a conservation network when its AOO is > 50 grid cells. Fixing the adequacy target for widely distributed taxa to 50 grid cells ensures representing > 10% of the AOO for taxa having the largest AOO in the dataset used (421 grid cells). The analysis steps were as follows.

1. Select taxa with AOO ≥ a threshold (T) (*LtoS*) or ≤ T (*StoL*)
2. Identify efficient set of grid cells that meet the targets of all the taxa selected in step 1 using the Marxan algorithm^34^.
3. Record the number of presence records used in step 2, the total cost of selected grid cells, and the number of taxa meeting the target given the conservation network identified in step 2.
4. Repeat steps 1–3 through T = 1 to 421 (*StoL*) and = 421to 1 (*LtoS*), respectively.

To estimate the relative cost of conducting surveys in grid cells for use as a cost layer in Marxan, we calculated the product of proportion of urban area and number of farmers within each grid cell^35^, under the assumption that an increase in the number of farmers and urban landowners increases the difficulty of obtaining permission for the surveillance^36^. The former was calculated from the land use subdivision mesh of 2009 (http://nlftp.mlit.go.jp/ksj-e), and the latter was obtained from the national population census of Japan in 2005 by Statistics Japan^37^, in each case divided by the maximum value and adding 1 to standardize range of the value between 1 and 2 and avoid multiplying by zero. Standardization was done to avoid the prioritization results being mainly determined by distribution pattern of cost, cf.^29^, because our focus is to explore general rules rather than identifying priority areas for implementation. Although the standardization influenced the cost curve, and thus could affect results of the prioritization, its influence was small for our results, because similar patterns were obtained on simulated data using constant cost across the space (see below for simulation analyses). We did not consider the spatial configuration of prioritized areas. In each Marxan run in step 2, we generated 100 solutions and evaluated both the number of overall taxa meeting the target, and the sum of the cost of selected grid cells for each solution, to obtain minimum, median, and maximum values of both of the measures, respectively. We used only the trend in median value of the number of taxa meeting the target, and that in the median value of the total cost of selected grid cells, respectively. We iterated the *random* strategy 50 times, and then obtained a lowess smoothed trend of the number of taxa meeting the target as the number of records used increased, and the total cost of the network as the number of taxa meeting the target increased. We used *loess* function in R 4.0.0^38^ to obtain the smoothed trend.

Based on above results, we quantified the magnitude of the informativeness of *StoL* over *LtoS* on the number of taxa meeting the conservation targets per number of records used and per total cost of selected grid cells, respectively, in comparison with the informativeness of *random.* For quantification, we define Relative Area Below the Curve (RABC): RABC uses area under the curve achieved by respective strategy, and shares its basic concept with area under the curve (AUC)^39^, Species accumulation index (SAI) of Albuquerque and Beier^10^, and Water Residence Time (WRT) of Hermoso et al.^40^ in using area below the curve to quantify its performance. We calculated RABC as follows:

$${\text{RABC}}=\frac{[({\text{the area under }}StoL)/({\text{the area under }}StoL +{\text{the area under }}random)]}{[({\text{the area under }}LtoS)/({\text{the area under }}LtoS +{\text{the area under }}random)]}$$

Numerator and denominator of the formula represent performance of *StoL* and of *LtoS* in comparison to the performance of *random,* respectively, and thus RABC represent relative performance of *StoL* over that of *LtoS*. For example, a value of 2.0 indicates that *StoL* is 200% more efficient than *LtoS* in the performance, in comparison to the performance of *random*. By definition, the RABC of r*andom* is always 1 (the ratio of 0.5–0.5). The number of records required to meet a conservation target of 75% of the overall taxa was assessed based on the relationship between total number of taxa meeting a conservation target and the number of records used to identify conservation networks, respectively for both conservation targets and taxa orderings.

We further conducted a simulation to confirm that observed informativeness of *StoL* over *LtoS* in our dataset is independent of the pattern of overlaps in taxon distributions or the shape of the study area. We created 1630 ‘virtual’ taxa each having a single patch randomly in a square space on 68 × 68 grid cells (i.e. approximately similar to the total number of grid cells on the study region), and assessed the performance on *StoL* over *LtoS* following the same procedures on the empirical distribution data. The AOO of each virtual taxa was drawn from a log-normal distribution of which mean and standard deviation were approximated from the empirical threatened taxa data (mean = 9.24, sd = 21.02). Additionally, we generated further sets of virtual taxa from the same distribution and mean but with threefold standard deviation of the empirical approximation (9.24, 63.06) to assess whether our use of threatened taxa (i.e. lack of taxa having very large AOO) do not contradict with our empirical findings. We generated 50 sets of virtual distributions for each set of parameters and assumed for the simulation that conservation costs are uniform across the virtual space. Integer linear programming could provide exact solutions^41,42^, however, Marxan is widely used among planners, and we consider that changing the method would have influence little on our result. This is because preliminary analysis of a range of different Marxan parameter settings, which differ in the extent to which the solution space is searched, resulted in only very small differences to results (Supplementary Information 2).

To explore underlying mechanisms driving the informativeness of narrowly distributed taxa, we assessed the relationship between taxon AOO and taxon richness using quantile regression using the empirical data (function *rq* in package *quantreg* run in R4.0.0^38^). We used mean taxon richness of grid cells in which respective taxon was observed as a response variable and estimated the relationship using data between the 10th and 90th percentiles at intervals of 10%.

## Supplementary Information

Supplementary Information 1.

Supplementary Information 2.

Supplementary Information 3.

Supplementary Information 4.

Supplementary Information 5.

Supplementary Information 6.
