# Supplementary material for: Narrowly distributed taxa are disproportionately informative for conservation planning
Source: Sci Rep. 2022 Feb 9;12:2229. doi: 10.1038/s41598-021-03119-9 (PMC8828766; doi:10.1038/s41598-021-03119-9)
Supplement: Supplementary file 2 — Supplementary Information 2. [file 41598_2021_3119_MOESM2_ESM.docx]

**Narrowly distributed taxa are disproportionately informative for conservation planning**

Authors: Munemitsu Akasaka, Taku Kadoya, Taku Fujita, Richard A. Fuller

**Supplemental material 2.** Parameter settings on Marxan analysis

We adopted parameter settings shown in Table S2-1 throughout all the Marxan analyses. Parameters used were determined following Ardron et al (2020). Parameter calibration was done for both empirical data and simulated data. For calibration on empirical data, we used distribution data of all 1630 taxa. For the virtual taxa, we generated 4 sets of 1630 virtual taxa to calibrate simulated data, respectively for the two parameter settings to generate virtual taxa (i.e., simulated data 1SD, and simulated data 3SD). To determine SPF, we run Marxan analysis on 4 candidate values (2, 10, 100, 1000, and 10000) on the all the combination of the two conservation targets (i.e. representation target and adequacy target) and on the all data type (1 empirical and 2 simulated). Solution with species penalty factor (SPF) = 2 performed best for the representation target regardless of the data type (Fig. S2-2a, c, e), and the performance of the solutions was not largely different across 4 candidate values on adequacy target on all data types (Fig. S2-2b, d, f). We therefore adopted SPF = 2 in the main analysis throughout. Number of iterations in the simulated annealing, and temperature decreases were set to default (1000000, 10000, respectively). Although increase in the former improved performance of Marxan solution on empirical data analysis (Fig. S2-3), it had little effect on our final measurements (Fig. S2-4). We therefore used the default value considering necessary time to complete the analysis (approximately 50 hours to finish one iteration of the simulation in our environment (CPU of the work stations: Intel i7-6700K or AMD Ryzen Threadripper 2990WX) even when the default value was used). Repeat runs of Marxan was set to 100 (default), because Marxan irreplaceability based on 100 runs and on 200 runs was highly consistent, regardless of the conservation target and data type (Fig. S2-5).

Although we calibrated the parameters stated above, difference in the parameter setting likely influence little on the overall pattern — in addition to the limited effect of number of iterations of simulated annealing on our measurements on the empirical data(Fig. S2-4), change in SPF value made little difference (Fig S2-6).

Ardron JA, Possingham PH, and Klen CJ 2010 Marxan Good Practices. Handbook, Version 2. Pacific Marine Analysis and Research Association, Victoria, BC,. Canada. 165 pages.

| **Table S2-1.** Parameter setting on Marxan analysis. | |
| --- | --- |
| Parameter | Value |
| SPF | 2 |
| Run mode (Algorithm) | Simulated annealing only  (with adaptive annealing) |
| Number of iterations on simulated annealing | 1000000 |
| Temerature decrease in simulated annealing | 10000 |
| Repeat runs | 100 |
|  |  |

**Fig. S2-2**
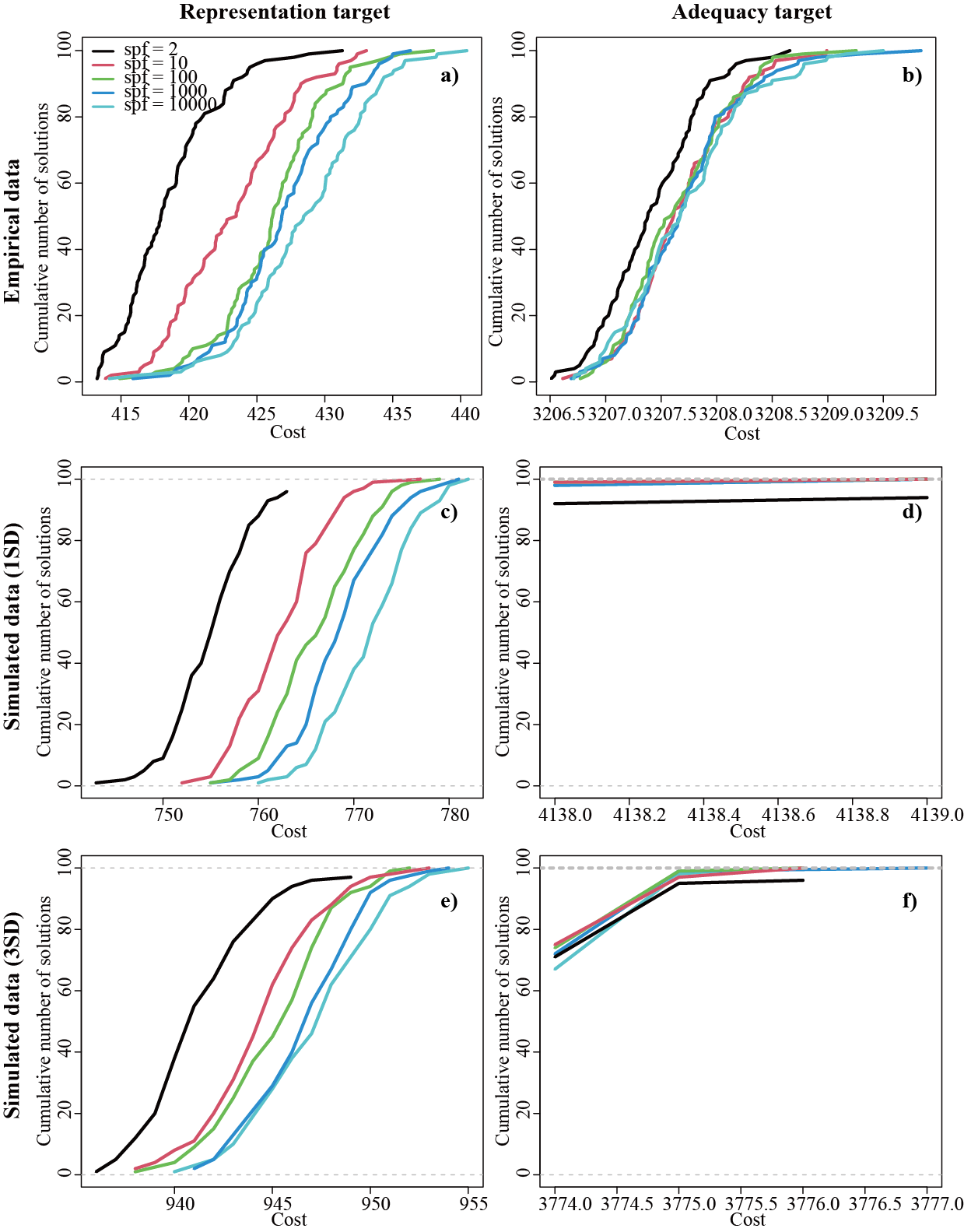
. Difference in performance of Marxan solution with different species penalty factor (SPF) across 3 data types and 2 conservation targets.


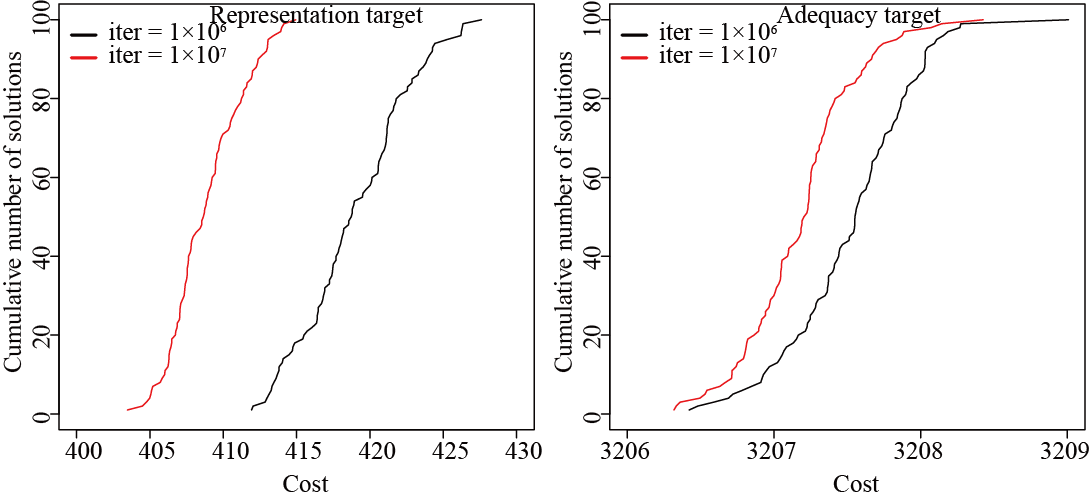


**Fig. S2-3.** Difference in performance of Marxan solution with different number of iterations in the simulated annealing on empirical data.


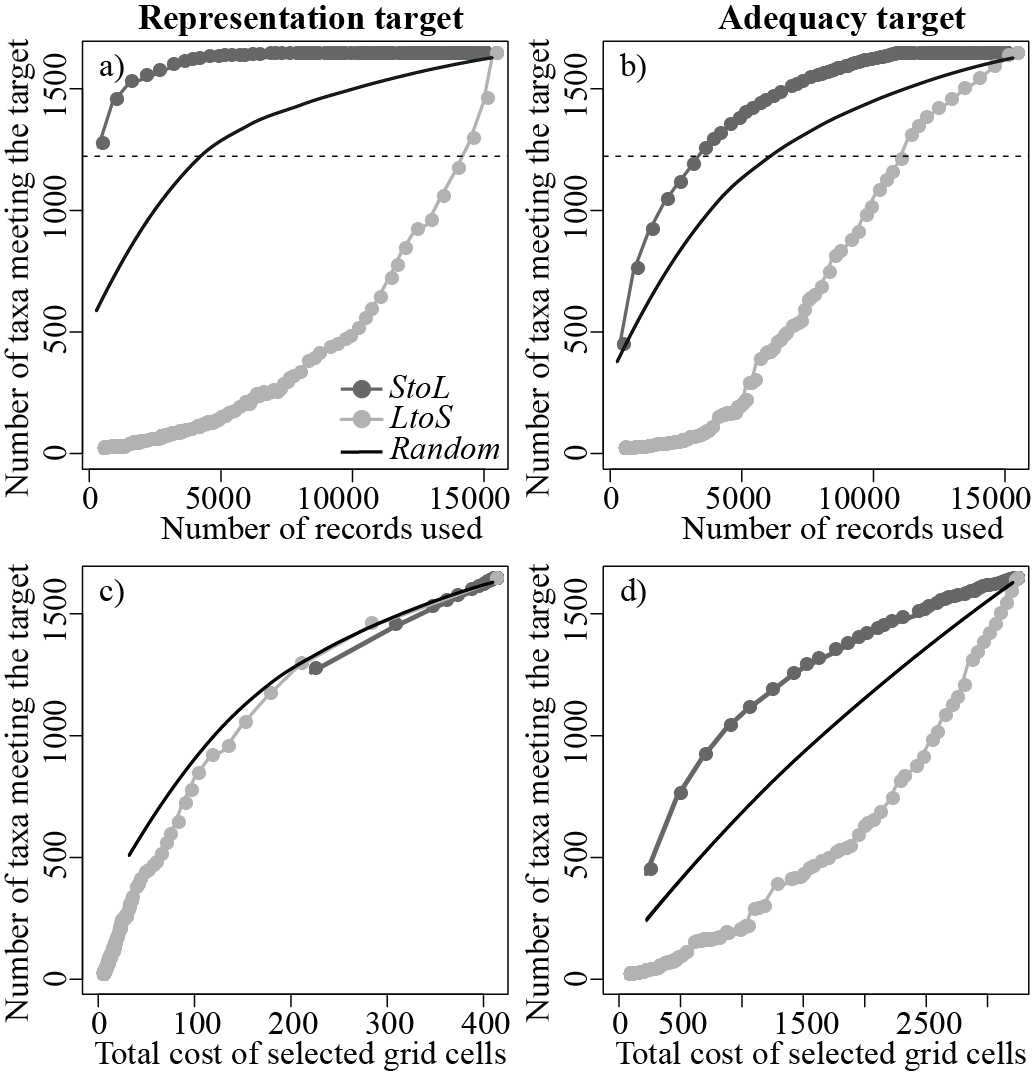


**Fig. S2-4.** Results of the analysis on empirical data adopting the number of iterations in the simulated annealing = 1×10^7^. See Fig. 1 for the results adopting the number of iterations in the simulated annealing = 1×10^6^.


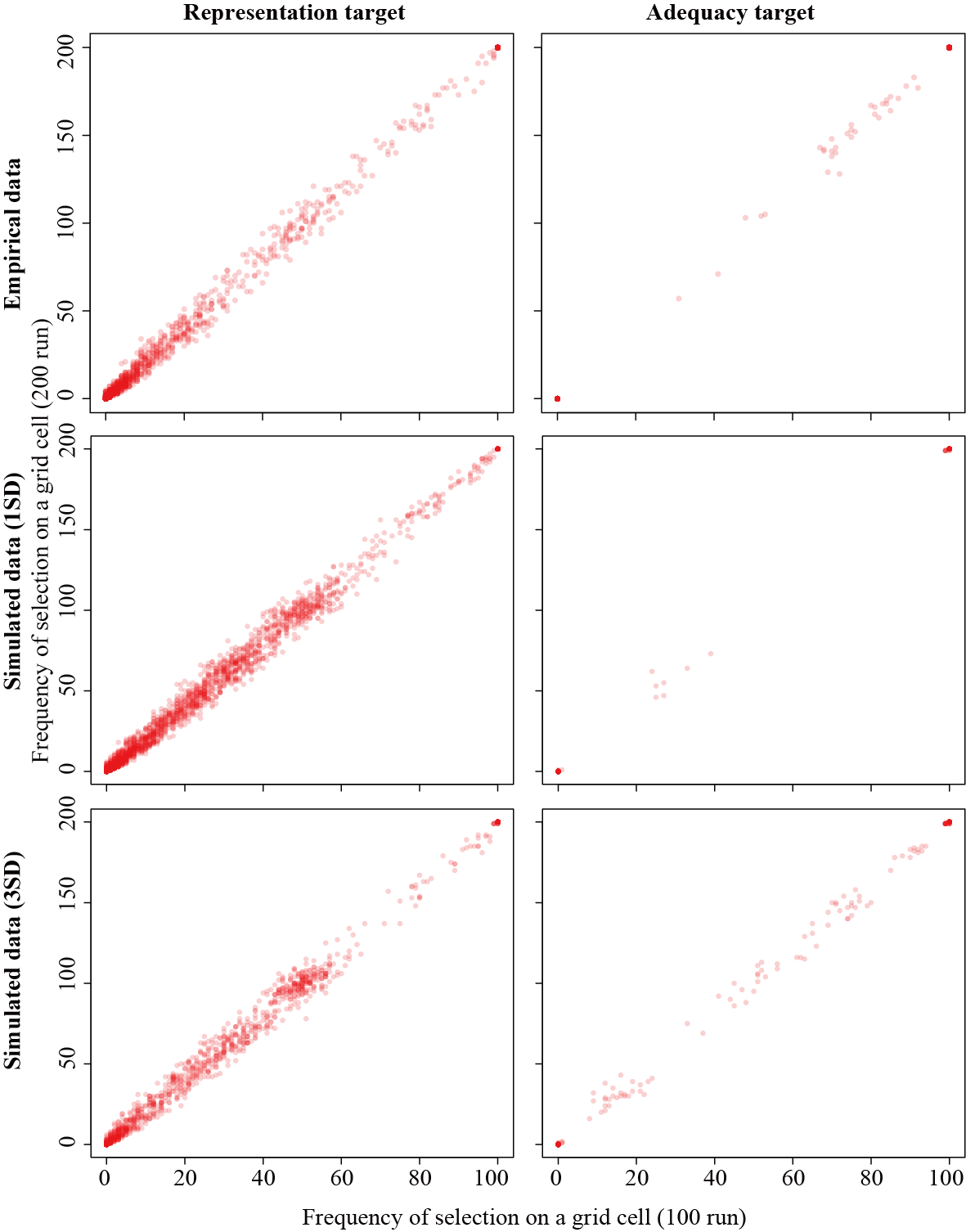


**Fig. S2-5.** Relationship between frequencies of selection on gird cells based on 100 and 200 Marxan runs.

**
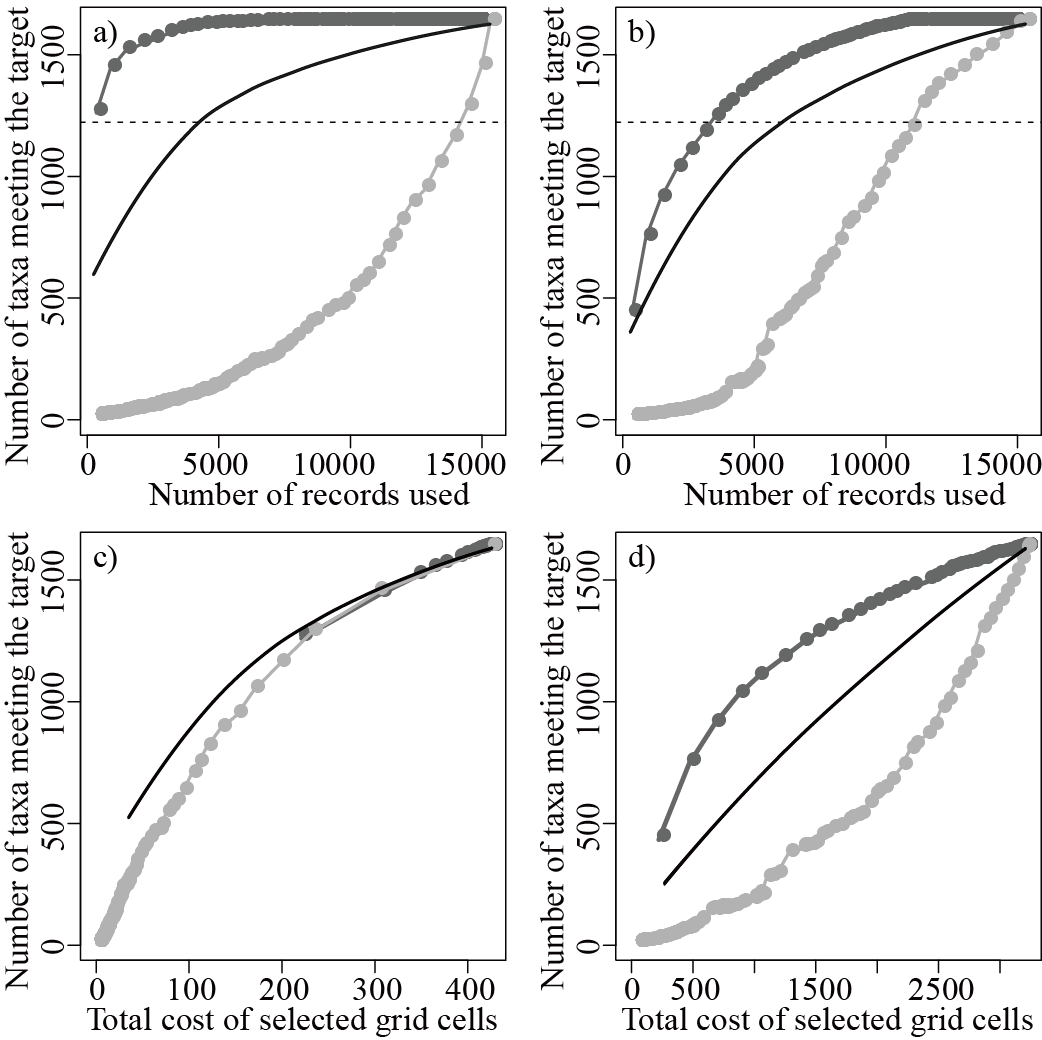
**

**Fig. S2-6** Results of the analysis on empirical data adopting species penalty factor (SPF) = 10, while remaining parameters were same to Table S2-1. See Fig. 1 for the results adopting SPF = 2.
