# Supplementary material for: Narrowly distributed taxa are disproportionately informative for conservation planning
Source: Sci Rep. 2022 Feb 9;12:2229. doi: 10.1038/s41598-021-03119-9 (PMC8828766; doi:10.1038/s41598-021-03119-9)
Supplement: Supplementary file 3 — Supplementary Information 3. [file 41598_2021_3119_MOESM3_ESM.docx]

**Narrowly distributed taxa are disproportionately informative for conservation planning**

Authors: Munemitsu Akasaka, Taku Kadoya, Taku Fujita, Richard A. Fuller

**Supplemental material 3**

| Supplemental table 3-1. Area under the curve of respective trends on Figure 1. | | | | |  |  |  |  |
| --- | --- | --- | --- | --- | --- | --- | --- | --- |
|  |  | Number of taxa meeting the target vs Number of records used | | |  | Number of taxa meeting the target vs Total cost of selected grids | | |
|  |  | *StoL* | *random* | *LtoS* |  | *StoL* | *random* | *LtoS* |
| Empirical data | |  |  |  |  |  |  |  |
|  | Representation target | 160002.7 | 133335.7 | 46597.43 |  | 102428.1 | 116135.6 | 108152.6 |
|  | Adequacy target | 141486.5 | 123496.2 | 72879.57 |  | 120225.2 | 95108.26 | 57486.22 |
| Simulated data | |  |  |  |  |  |  |  |
|  | Representation target | 157276.33 ± 463.56 | 110294.81 ± 457.46 | 40401.31 ± 1315.46 |  | 91200.66 ± 684.76 | 95631.8 ± 376.47 | 98516.41 ± 581.35 |
|  | Adequacy target | 136595.45 ± 1345.21 | 107052.17 ± 680.4 | 74785.96 ± 3178.66 |  | 98887.5 ± 867.93 | 70376 ± 681.08 | 58462.46 ± 1100.61 |
