# Supplementary material for: Narrowly distributed taxa are disproportionately informative for conservation planning
Source: Sci Rep. 2022 Feb 9;12:2229. doi: 10.1038/s41598-021-03119-9 (PMC8828766; doi:10.1038/s41598-021-03119-9)
Supplement: Supplementary file 4 — Supplementary Information 4. [file 41598_2021_3119_MOESM4_ESM.docx]

**Narrowly distributed taxa are disproportionately informative for conservation planning**

Authors: Munemitsu Akasaka, Taku Kadoya, Taku Fujita, Richard A. Fuller


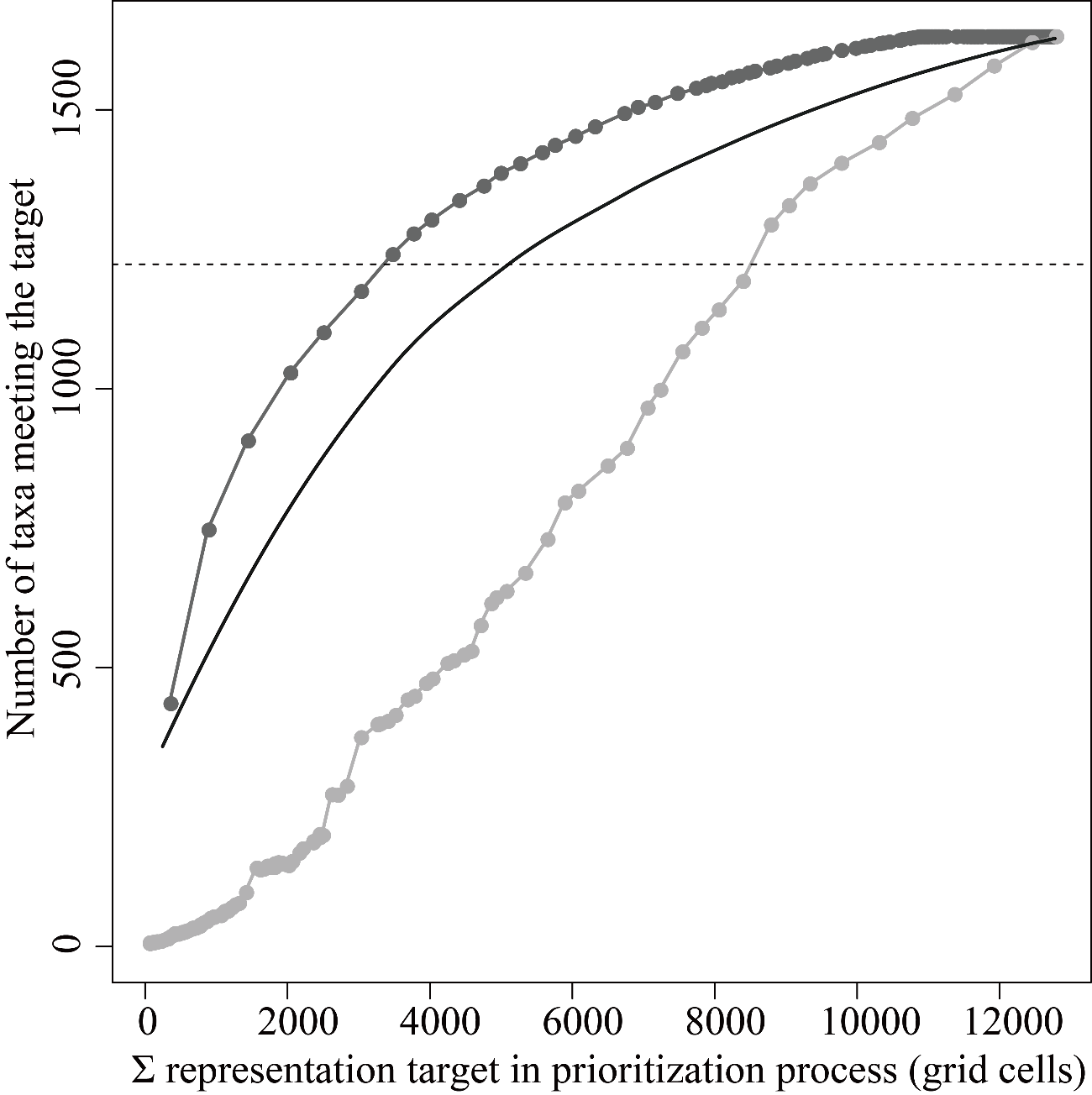


**Supplemental material 4.** Relationship between sum of the representation target used to select grid cells (*x*-axis) and the number of taxa meeting conservation targets (*y*-axis) using taxa distribution data sequentially from small to large (*StoL*: dark grey), large to small (*LtoS*; light grey) or a random order (black). For the random order, the relationship was displayed by drawing a lowess smoothing line of the 50 iterations. RABC = 1.277.
