# Supplementary material for: Narrowly distributed taxa are disproportionately informative for conservation planning
Source: Sci Rep. 2022 Feb 9;12:2229. doi: 10.1038/s41598-021-03119-9 (PMC8828766; doi:10.1038/s41598-021-03119-9)
Supplement: Supplementary file 5 — Supplementary Information 5. [file 41598_2021_3119_MOESM5_ESM.docx]

**Narrowly distributed taxa are disproportionately informative for conservation planning**

Authors: Munemitsu Akasaka, Taku Kadoya, Taku Fujita, Richard A. Fuller

**Supplemental material 4**

**
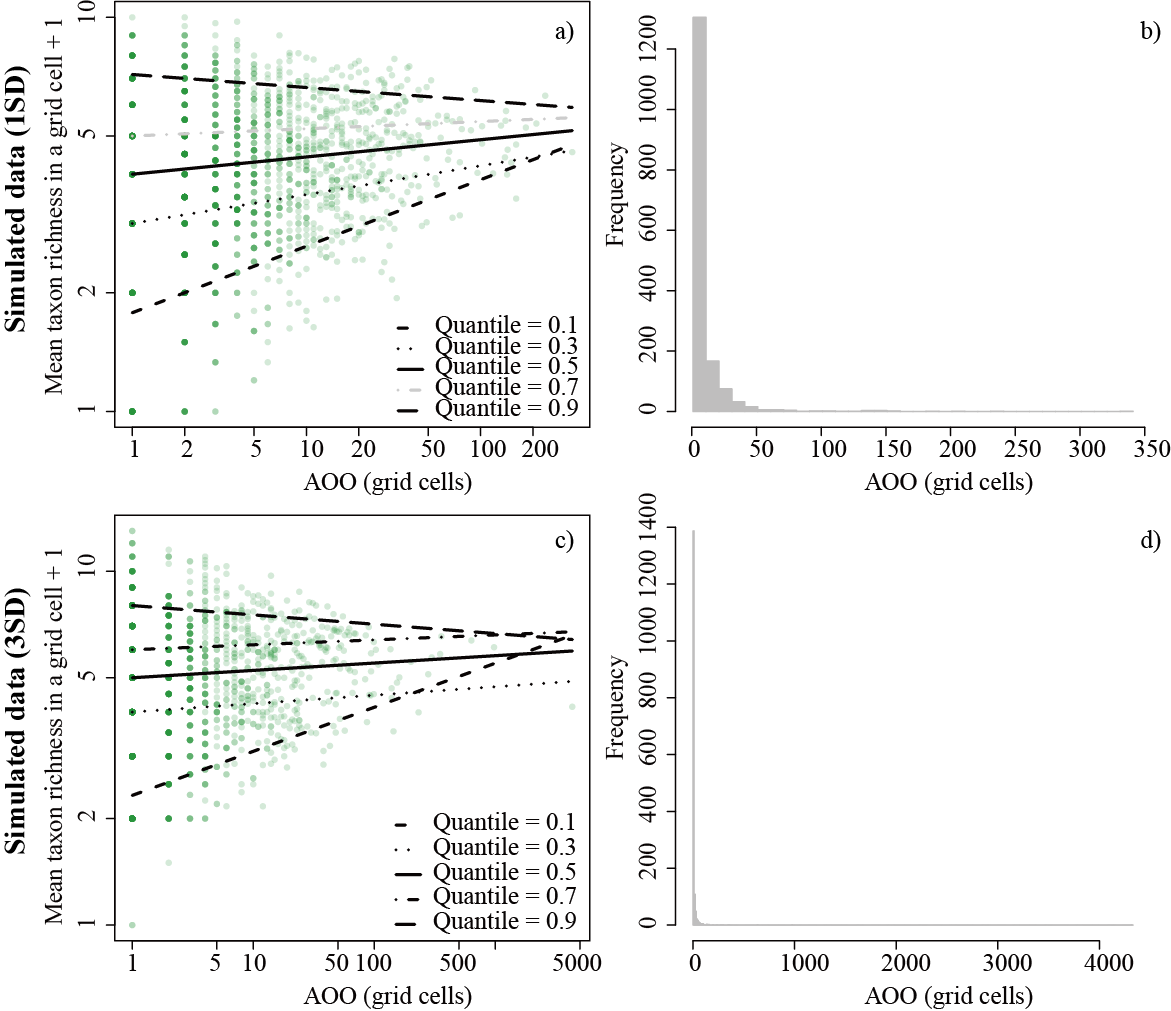
**

**Supplemental figure 4-1**. Examples of the relationship between taxon richness per grid and species’ AOO for virtual taxa generated based from a log-normal distribution with a) mean = 9.24 and sd = 21.02, and c) mean = 9.24 and sd = 63.6. A histogram of b) AOO on taxa pool used in a), and of d) AOO on taxa pool used in c).
