# Supplementary material for: Narrowly distributed taxa are disproportionately informative for conservation planning
Source: Sci Rep. 2022 Feb 9;12:2229. doi: 10.1038/s41598-021-03119-9 (PMC8828766; doi:10.1038/s41598-021-03119-9)
Supplement: Supplementary file 6 — Supplementary Information 6. [file 41598_2021_3119_MOESM6_ESM.docx]

| **Supplemental table 6.** Estimates and SE of quantile regressions between taxa’ AOO (explanatory variable) and mean frequency of overlapped taxa per grid (response variable). Both variables were log transformed prior to the regression analysis. A value of 1 was added to the response variables before log-transformation to avoid 0. * p < 0.00001, NS: not significant. | | | |
| --- | --- | --- | --- |
|  |  | Estimates | Intercept |
| Quantile = 0.1 | |  |  |
|  | Estimate | 0.113* | 1.386* |
|  | SE | 0.023 | 0.063 |
| Quantile = 0.2 | |  |  |
|  | Estimate | 0.058* | 1.792* |
|  | SE | 0.012 | 0.044 |
| Quantile = 0.3 | |  |  |
|  | Estimate | -0.015^NS^ | 2.15* |
|  | SE | 0.012 | 0.044 |
| Quantile = 0.4 | |  |  |
|  | Estimate | -0.084* | 2.485* |
|  | SE | 0.016 | 0.052 |
| Quantile = 0.5 | |  |  |
|  | Estimate | -0.154* | 2.816* |
|  | SE | 0.013 | 0.056 |
| Quantile = 0.6 | |  |  |
|  | Estimate | -0.232* | 3.219* |
|  | SE | 0.015 | 0.054 |
| Quantile = 0.7 | |  |  |
|  | Estimate | -0.299* | 3.584* |
|  | SE | 0.009 | 0.045 |
| Quantile = 0.8 | |  |  |
|  | Estimate | -0.355* | 3.951* |
|  | SE | 0.020 | 0.038 |
| Quantile = 0.9 | |  |  |
|  | Estimate | -0.39* | 4.347* |
|  | SE | 0.030 | 0.073 |

**Narrowly distributed taxa are disproportionately informative for conservation planning**

Authors: Munemitsu Akasaka, Taku Kadoya, Taku Fujita, Richard A. Fuller
